# Multi-layer laser cutting of electrical steel sheets applied to electric machine laminations

**DOI:** 10.1371/journal.pone.0288232

**Published:** 2023-07-07

**Authors:** Nathan Dodd, Erica Ballantyne, Graeme Heron, Russell Goodall

**Affiliations:** 1 Department of Mechanical Engineering, The University of Sheffield, Sir Frederick Mappin Building, Sheffield, United Kingdom; 2 Sheffield University Management School, The University of Sheffield (UK), Sheffield, United Kingdom; 3 Industrial Doctoral Centre in Machining Science, Advanced Manufacturing Research Centre with Boeing, University of Sheffield, Rotherham, United Kingdom; 4 Dept Materials Science & Engineering, The University of Sheffield, Sir Robert Hadfield Building, Sheffield, United Kingdom; University of Sharjah, UNITED ARAB EMIRATES

## Abstract

With the move away from fossil fuels, the importance of electric machines is increasing. This is particularly the case within major engineering sectors such as the automotive industry. There is therefore a need to further develop processes which will allow for the diverse range of machining operations and large volume manufacture which will be required to overcome the inherent challenges in making this transition. Several critical components of an electric machine, such as the rotor and the stator, are made from electrical grade steel. This is a steel where the composition and processing acts to optimise the magnetic and other properties for the application. The steel is processed as thin sheet laminations and then stacked, to reduce the losses which occur within it due to the generation of eddy currents. The laminations need to be cut to shape, in an operation currently carried out most frequently by stamping from a sheet, but which could be done with greater flexibility by laser cutting (due, for example, to the absence of tooling). In laser cutting the possibility exists to perform cutting operations using what we call here a *polystromata* method, where several sheets are stacked and then cut simultaneously, increasing the efficiency of the operation. To date there have been few reports on this type of laser cutting process, and none that provide detail on the effect that the number of layers in a cutting stack has on critical parameters, such as the edge quality post cutting and the magnetic performance of the sheets. In this work we perform an experimental study of the process and report data in these measures, quantifying the decrease in performance as the stack increases in number of sheets.

## 1 Introduction

Advances in manufacturing technologies are typically implemented due to the effect of raising the quality of products produced or enabling a reduction in the cost of production. In the automotive industry, electric machines (or motors) have thin metal sheet components which are currently manufactured by stamping, which has been the case for a number of years [1, 2]. Other approaches to shaping these sheets (such as that studied specifically herein, laser cutting) commonly appear at early stages of development to be economically unviable due to the slower manufacturing times [3]. As a result of this mindset little research has been committed to advance these technologies towards greater mass manufacturing viability. For new laser cutting methodologies, as explored in this work, to further the drive towards superior and more efficient electric machines it is essential to develop a greater understanding of the effect that the manufacturing processes have on materials and components.

An electric machine is made from four key components: stator, rotor, winding and shaft. The stator and rotor are generally made from electrical steel sheets and are typically stamped. The use of laser cutting to manufacture components such as stators and rotors is an area of production strategy which has remained undeveloped due to the established use of stamping and the perceived technological limitations from a manufacturing efficiencies perspective. Stamping requires a high level of initial investment owing to the manufacture of dies, but once set up, is a relatively quick and consistent method. Laser cutting also demands high initial investment, and is a much more time-consuming process, but offers vastly superior flexibility and as such is a preferred method for producing small batches or one offs [1]. Whilst other cutting processes exist, such as abrasive water jet and wire cut electric discharge machining, these are typically used for specific applications, rather than in large scale manufacture [2]. Recent studies have concluded that controlling the cutting process is important to achieve a high surface quality without sacrificing magnetic properties [3].

The economic efficiency of laser cutting can be significantly increased by performing identical cuts on several layers simultaneously within a single cutting operation by stacking the layers [4]. This process of stacking laminates prior to laser cutting is herein referred to as the *polystromata* method, due to the multi-layer nature. While the approach gives an evident increase in processing efficiency, it is unclear how the quality of parts produced in this way might be affected. Stamping is a mechanical process, but laser cutting is a thermally intense process and, as such, the effects of laser cutting differ somewhat from stamping. Changes to the microstructure of the material can have potentially negative consequences for the magnetic performance of the material, with effects such as recrystallization texture and grain size change being potential causes for performance loss [5].

This paper seeks to understand the challenges and outcomes in terms of the material structures and properties of manufacturing parts using the polystromata method, which offers cost advantages compared to conventional laser approaches, and appears to be an economically viable alternative to stamping. The research undertakes experimental trials of multi-layer laser cutting on electrical steel laminations and analyses the effect of the of laser cutting process on the material after cutting. The results from these studies are used to compare the quality of polystromata laser cut samples to similarly stamped sheet material and also to understand the condition and quality of samples at different positions within a polystromata stack cut, hence setting upper limits on how large a stack can be produced in this embodiment of the process.

## 2 Background

Stamping is an established process for producing shaped laminations. Bayraktar & Turgut [6] and Jayarama [7] both investigate the effect of stamping on the quality of the cut edge. M400-50A (Bayraktar & Turgut) and HYPERCO 50 (Jayarama) are the materials tested. In these trials the evidence suggests that stamping does not always generate a clean, full shear cut. These results also demonstrate some deformation and rounding in the upper region of the cut.

One of the main competitor processes to stamping for this application is laser cutting. A laser works by focusing a high intensity light source into a very narrow beam. The laser beam is amplified by passing through a medium which can be either a solid, such as a crystal, or an assist gas [8]. The high energy of the laser beam melts the workpiece at the contact point, and this action of melting away material creates the cut in laser cutting. Separate to the assist gas there is an auxiliary gas which blows away molten material from the workpiece. The auxiliary gas can be inert or active. An inert auxiliary gas protects the surface from oxidation, whereas an active gas (usually oxygen) generates an exothermic reaction at the workpiece, which increases the temperature at the cutting area, allowing thicker cuts to be produced [9]. Laser cutting is capable of dimensional tolerances in the region of 0.01mm [10].

Gaworska-Koniarek [11] has studied the effect of using different cutting gases on the electromagnetic performance of electrical steels. Gaworska-Koniarek concluded that the use of compressed air had a more detrimental impact on the magnetic properties of the sheet than cutting with a nitrogen atmosphere. Gaworska-Koniarek proposes that the reduction in electro-magnetic performance may be as a result of iron oxides forming on the cut edge, although it appears unlikely that these would penetrate very deeply into the material. Gaworska-Koniarek also compared laser cut samples with punched samples, with results showing that for 1.5T induction, punched samples had total energy losses of 5% whereas for the samples laser cut with air the loss was 20% and samples laser cut with nitrogen showed 17% total energy losses. Salvador [12] has similarly compared the performance of laser cut and guillotined (a cutting method mechanically very similar to stamping) samples, again concluding that laser cutting introduces greater losses than stamping-like processes. Bayraktar & Turgut [6] compare four different cutting methods. On visual inspection of those results the stamped samples appear slightly better than the laser cut samples. The electromagnetic performance was measured, with a punched stator providing 85.16% motor efficiency and a laser cut stator 83.47%.

Krings [13] compared the performance of punched and laser cut nickel-iron samples after annealing. The results from that research show similar levels of performance between the stamped and laser cut laminates. Krings reports that the laser cut samples reach a slightly higher saturation flux density at the cost of a slightly larger magnetic coercivity and iron losses. Interestingly, Krings concludes that there is real potential to reduce iron losses by adjusting the manufacturing processes used, suggesting that some form of optimisation can be incorporated into the manufacturing and assembly processes to increase the quality of parts produced. With laser cutting being less established, it may be that there is more potential for optimisation leading to further improvement of the method than there is for stamping. Bali & Muetze [14] investigated the degradation effects of punching and laser cutting and showed that further research is required to understand effects that laser cutting has on the degradation of cut laminates, and the mechanisms by which these occur.

The research consistently demonstrates that laser cutting laminates introduces greater losses when used to produce stators for electric machines than punching laminates. In the best instances, the difference in performance is small, but not negligible. Miljavec [15] states that punching (or stamping) is the process which should be used, citing concerns that laser cutting will increase magnetic losses, but approves of the use of laser cutting in the production of prototypes. Research is beginning to be published which considers the effects of cutting sheets simultaneously in a single operation, noting further applications in automotive industry if the process can be sufficiently optimized [16]. The current research however does not consider the economic and wider operations effects of using laser cutting as an alternative manufacturing process, and the performance of the material that results from the operation is another critical factor which must be understood. This research reports on the results of an experimental study where we address the potential of cutting multiple laminates in one operation using laser cutting.

## 3 Method

**Fig 1:**
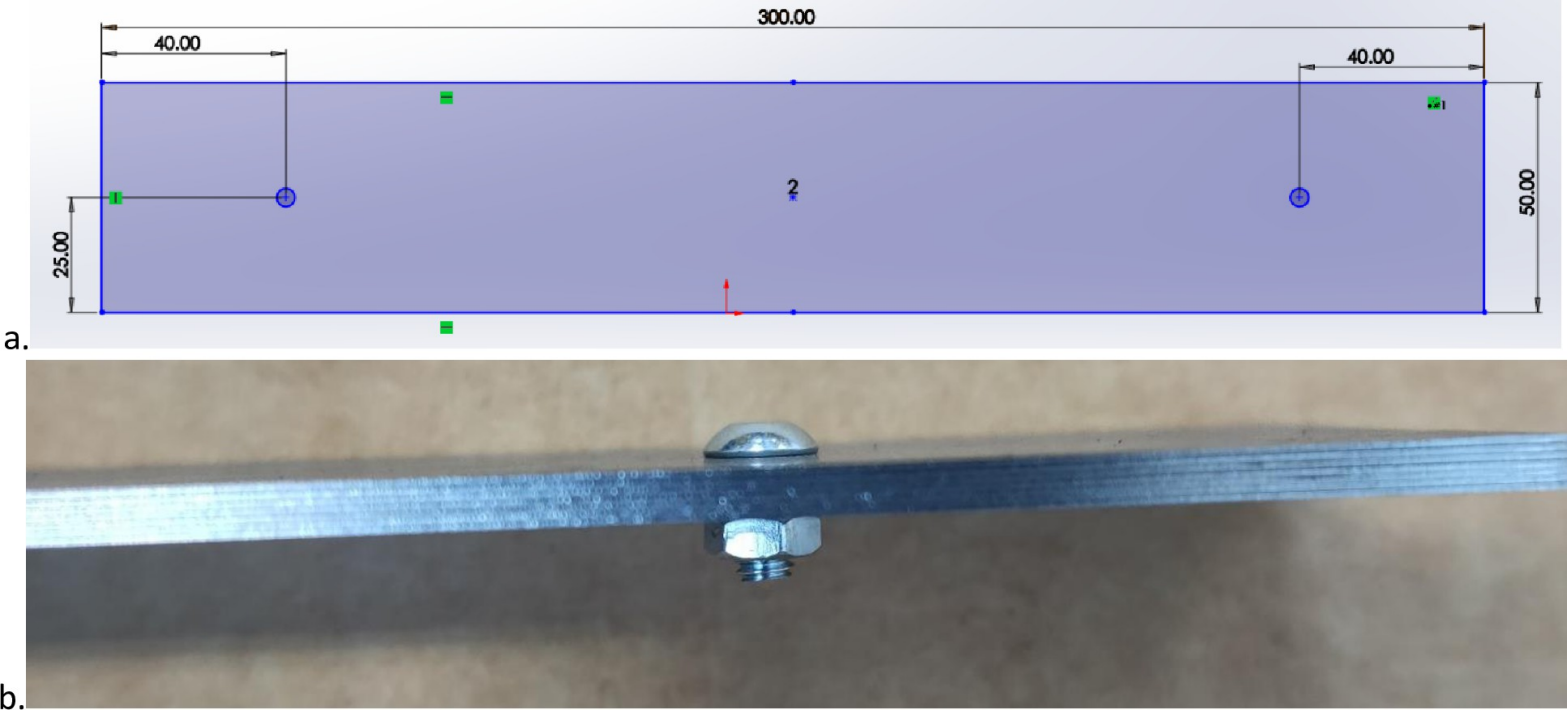
(a) Sample schematic [units in mm] (b) Side view of stack with bolt arrangement prior to cutting.

A polystromata cut (multi-layer stack of sheets, cut in a single operation) was performed by assembling sheets of 0.35mm thick Cogent M250-35A grade electrical steel into a 7-layer stack of material. Each stack was secured together using 4mm diameter bolts to reduce the potential of parts moving during cutting (Fig 1). The laser cutting trials were conducted using a 3kW Trumpf laser, located at the Advanced Manufacturing Research Centre, the University of Sheffield. In the operation, the laser beam penetrated through the 7-layer stack, simultaneously cutting each sheet. The laser used settings preset in that machine by the manufacturer. The values of the laser parameters cannot be accessed from the equipment, and as part of Trumpf’s intellectual property associated with their product, are not disclosed. The settings are identified as being intended for the cutting of 2.5mm thickness of copper, and were selected from the range of available presets for material and thickness after an initial trial study exploring the ability to cut the material (which was not always the case for lower thickness presets) and leave visually acceptable edge quality (which did not happen for some other material presets). Notably, presets corresponding more closely to the actual material (0.35mm thick electrical steel) were not found to be effective. The trial study also identified nitrogen assist gas as the best medium to use with the laser.

### 3.1 Scanning Electron Microscopy

After manufacture, Scanning Electron Microscope (SEM) examination was conducted to view the quality of the cut edge with greater detail by examining sections taken from the laser cut samples. Two full stacks were chosen at random for testing. Sections were taken from one end of each stack, and manually cut so that the samples could be mounted. To prepare the samples for microscopic examination, slices were adhered to each other using double sided conductive carbon tape and further wrapped in conductive copper tape. The assembly was held on an aluminium SEM stub with copper tape, providing a conductive pathway for the imaging electrons. For these examinations of sample edges, the specimens were unmounted, and no grinding, polishing or other preparation was used. The cut edge of samples was examined in an FEI Inspect F FEG-SEM at 15kV, recording images at magnifications ×100 and ×250 in secondary electron (SE) mode, using a spot size of 3.5 and aperture of 4. Where indicated, backscattered electron (BSE) images were also obtained to enhance contrast in regions of topographical and compositional difference.

Samples were also prepared and imaged to understand how the microstructure of the material had been affected as a result of the polystromata laser cutting process. The specimens were mounted edge-on in conductive Bakelite and ground and polished to a mirror finish (initially ground to P1200 grit paper, and then polished with 6μm and 1μm diamond, all performed on a Beuhler AutoMet 250 polishing machine). The exposed surface, which showed the cut edge on one side and then the section through the sheet, was imaged at ×500 magnification using both SE and BSE imaging in an FEI Inspect F FEG SEM at 15kV, Spot size 3/3.5 and objective aperture 5, examining the edge of the sample and a region roughly in the middle of each strip. The imaging mode allowed contrast between grains to be seen as predominantly channelling contrast, enhanced by a light etch (Nital) prior to examination to reveal the grain structure.

### 3.2 Epstein frame assessment of magnetic losses

A trial was conducted using the as-cut polystromata laser cut samples (in their full size of 300mm × 50mm). Samples were separated into those from layers 1–2, 3–4 and 5–6 (with the layers numbered from the top surface, which the laser beam strikes first). Layer 7 samples were not used because of non-conformance to the size requirements. Results were obtained based on these groupings of layers, testing samples according to the protocols provided by the machine suppliers, Laboratorio Elettrofisico [17]. The Epstein frame was set up to test at a frequency of 50 Hz and approximate flux density of Bv 0.303 T and magnetic polarisation of Jv 0.3 T for all trials.

## 4 Results

**Fig 2:**
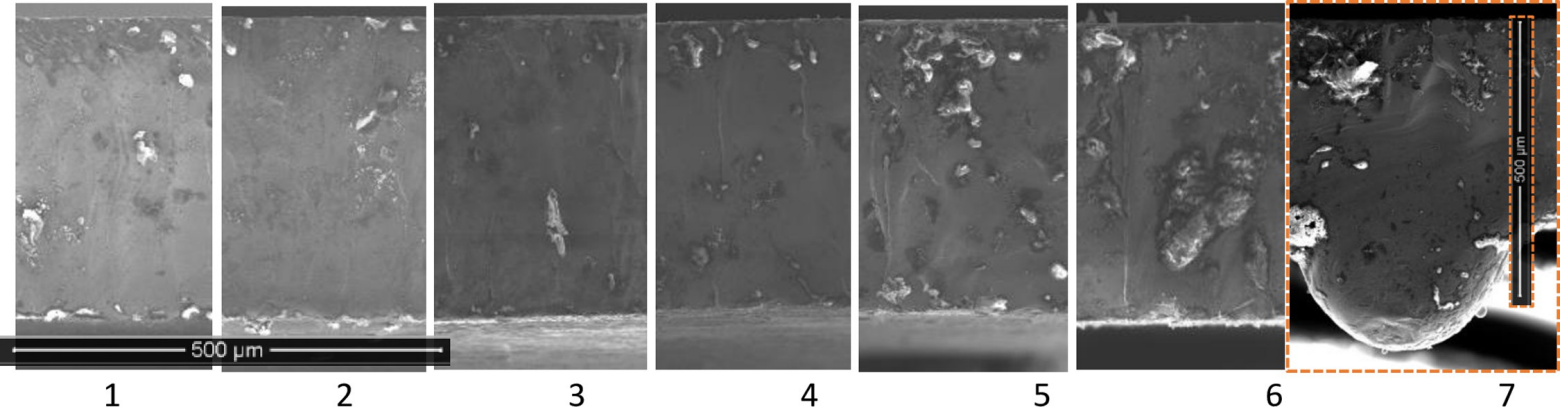
SEM results (×250 magnification) secondary electron 15kV for layers 1 (top) to 7 (bottom).

The results in Fig 2 show a consistent cutting pattern across layers 1–6. In these layers, there is negligible burring on the edges. Layer 7 in each stack clearly shows excess material on the underside of the layer. This is considered to be as a result of molten material which has collected either through dripping from layer 7 or being blown downwards between layers from those above. Layer 7 is not in a useable state to be included into a stator or rotor lamination stack without further processing to remove the excess material, an operation that is extremely unlikely to be efficiently possible. One solution would be to simply scrap layer 7, but this would mean scrapping 14.3% of material based on a 7-layer cutting stack. Assuming that only the bottom layer ever needs to be scrapped, the percentage of layers scrapped would be reduced by increasing the number of layers in the cutting stack. Alternatively, knowing that the final layer would be of insufficient quality, it would be possible to include a sacrificial sheet of a lower cost material as the final layer, though this would still represent some degree of additional wastage.

**Fig 3:**
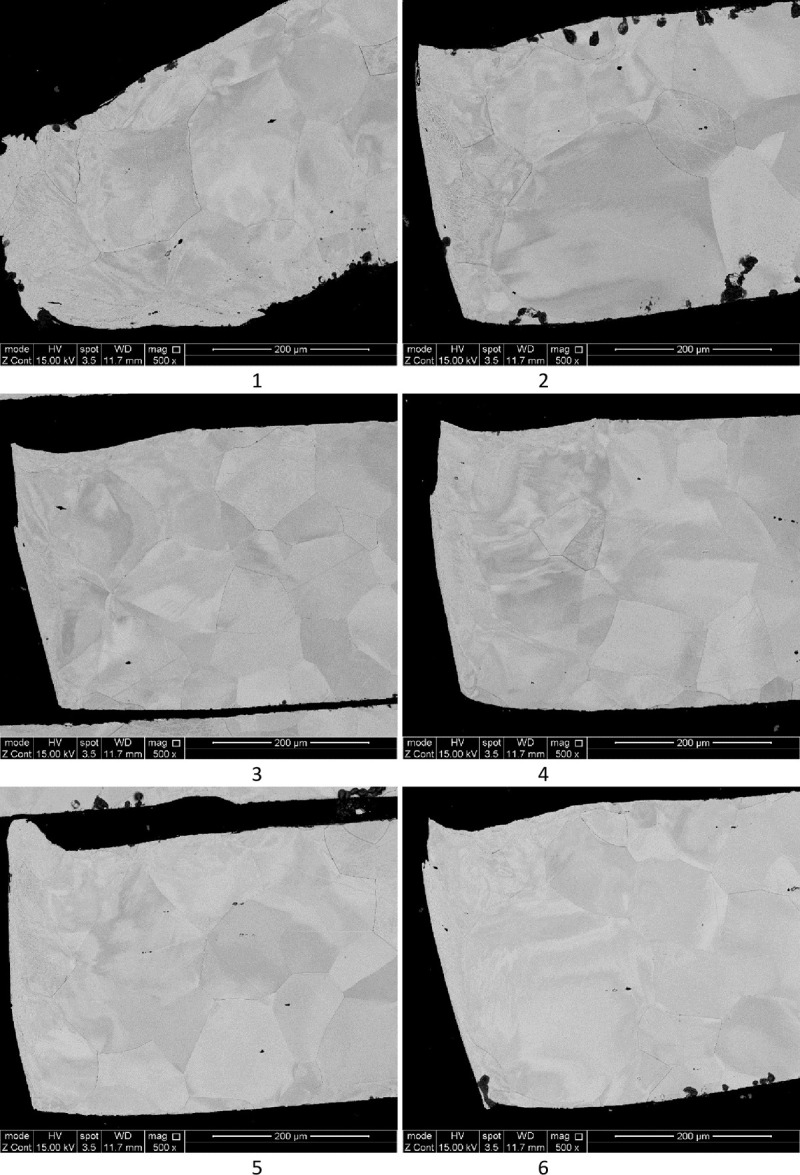
SEM results of cut edge (×500 magnification) secondary electron 15kV for layers 1 (top) to 6 (bottom).

A further investigation into the grain structure of the samples was performed with results shown in Figs 3 and 5. In all samples the area at the cutting edge shows a different material structure to the rest of the sample.

**Fig 4:**
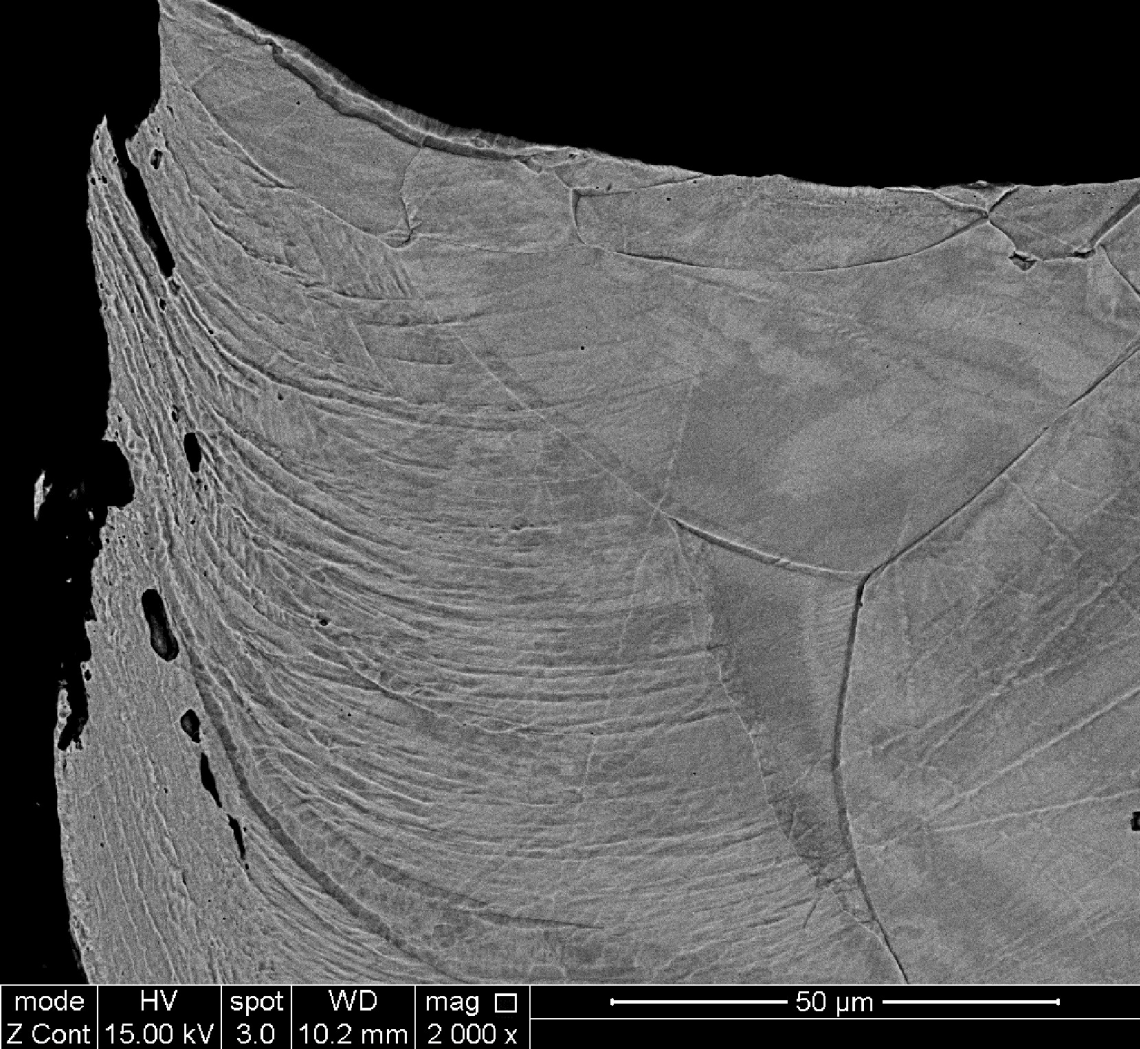
Stack 1, Layer 1. SEM ×2000 Zoom at cut edge.

The images in Figs 3 and 4 show a magnified cross section of the cut edge where laser penetration occurred form the top of the sample. Fig 4 shows a laminar structure, probably related to material flow or plastic slip at elevated temperatures, which penetrates into the material to a depth of approximately 50 μm on the upper section of the sample and 100 μm on the lower sections of the visible image. There is also consistent evidence that the cut edge is not ‘straight’, with a small burr occurring on most samples, and the cut receding into the material on the lower sections.

**Fig 5:**
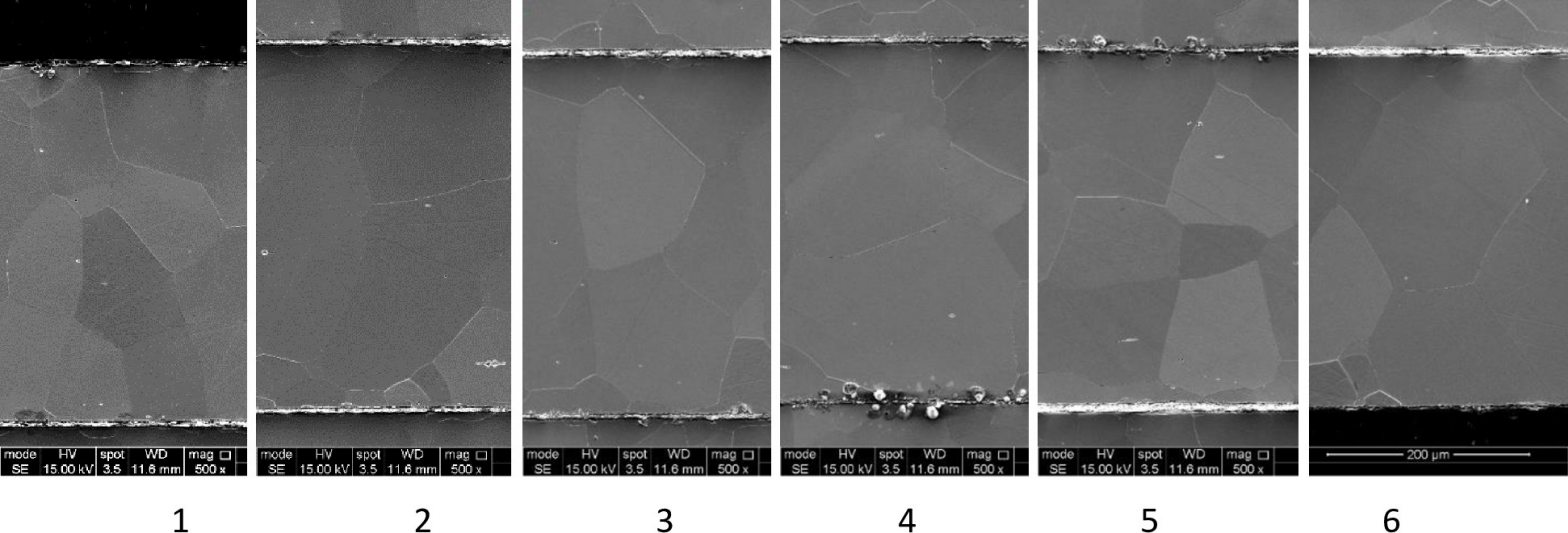
SEM results of mid-section (away from cut) (×500 magnification) secondary electron 15kV.

The mid-section images (Fig 5) show a largely consistent grain size through the material and between the layers, as would be expected for material that was not affected by the cutting process. There is also little apparent distortion of the grain size in the regions close to the cut, with grains of similar size to the bulk being found within 200 μm of the cut surface. This indicates that these areas are not exposed to sufficient heat for sufficiently long enough to cause grain growth, though as the initial grains are not fine, the driving force for grains to grow will not be very high.

### 4.1 Joined layers during polystromata cutting

**Fig 6:**
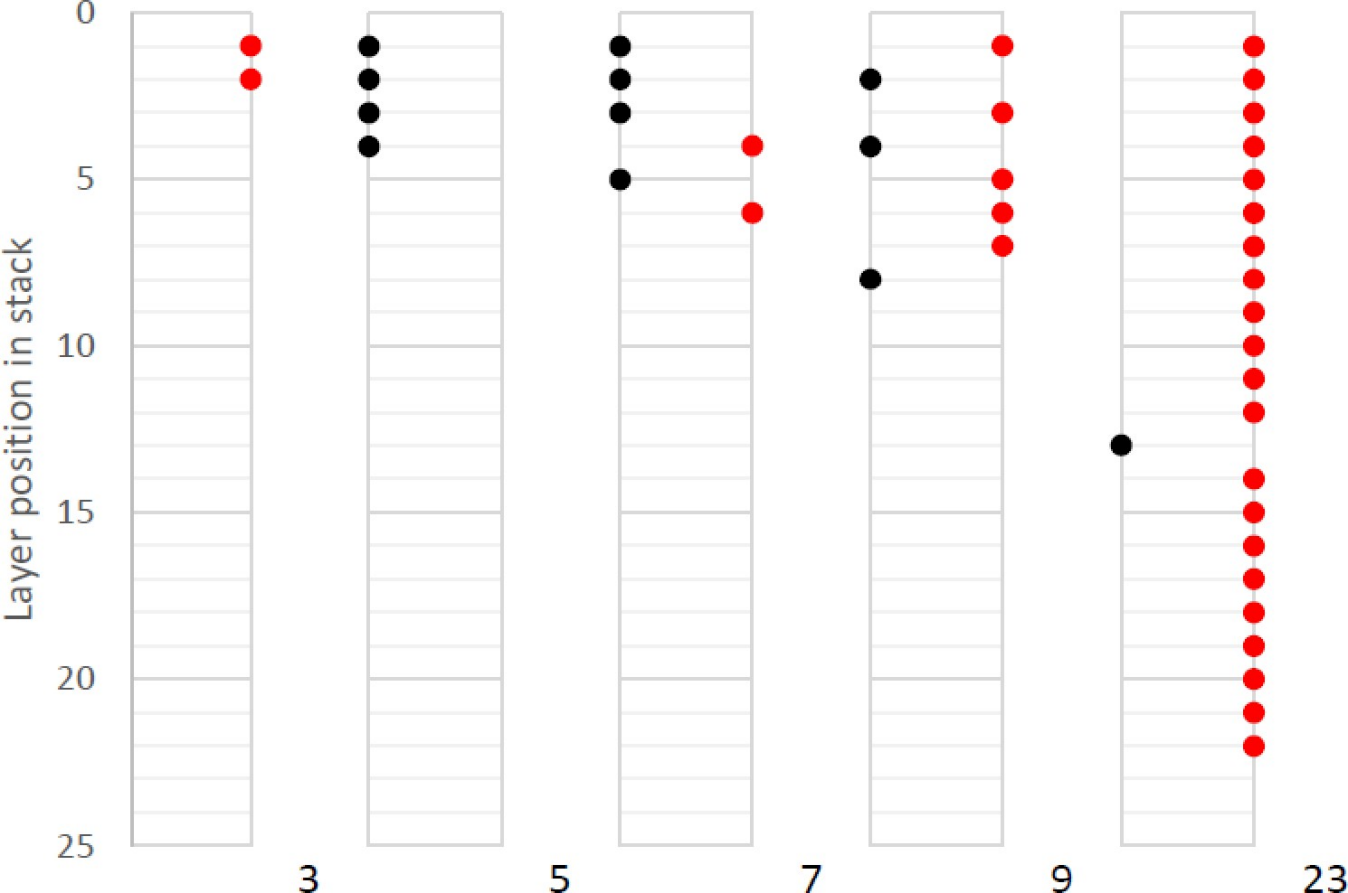
Layers joined in various laser cutting stack sizes (Black loose, Red joined to layer beneath).

During the stack cutting trials, it was seen that some layers within the stacks can become joined to each other, as the material at the edge is melted and flows down. A focussed trial was conducted to understand how this joining effect is influenced by stack size. The results of this trial are shown in Fig 6. The smallest stack tested containing 3 layers resulted in layers quite loosely joined together. A visual inspection appeared to show small droplets formed along the length of the cut. These droplets solidified in the gap between layers and effectively created a small weld. Separating these layers was easily manageable by hand without the use of high levels of force, though it would represent an additional step or process in a manufacturing system.

The 5-stack layer had no issues with layers being joined. However, there was evidence of a similar droplet pattern on the top of the cutting line of layer 3. Despite this, the layer was not joined to layer 2 (the layer above). Stacks of 7, from the initial cutting trial, and 9, were very similar. Some layers were joined, but the joins were easily separated by hand. The position of joins appears somewhat random, as some layers were joined close the laser start position, some along the length and some towards the laser end position. That said, there does appear to be a higher likelihood of joining occurring at positions where the laser speed changes, such as when starting, finishing, or turning a corner. While the exact control parameters used in the equipment to determine how the laser moves are not available, in these locations the laser would likely to be moving more slowly for a period of time, and may therefore melt a larger volume of material, which is more likely to flow down.

The 23-layer stack was a poor performer in all respects examined. The stack was highly deformed, with a high degree of bending in the stack. The cut stack separated in two parts, as layer 13 was the only layer to not be joined the layer beneath. This appeared to be due to some of the bending deformation which had occurred in the middle layers of the stack. The joined layers were not able to be separated manually, and not without resulting in plastic deformation in the material.

### 4.2 Polystromata laser cutting issues

An attempt was made to cut a 7-layer stack using a more powerful laser set-up. This was done by altering the laser machine settings to presets identified as those required to cut the equivalent of 3mm of copper material instead of 2.5mm. The laser cutting machine is limited by default to only use nitrogen shielding gas up to sheet thicknesses of 2.5mm. As such, this larger thickness allowed oxygen to be used instead. The resulting cutting operation had catastrophic consequences for the material, such that the operation was halted shortly after starting. During the cutting process, many sparks were emitted from the material, which was unlike the other samples which were produced. There was substantial damage to the material, post cutting which had been melted, forming a solid section at the melt point. The presence of the oxygen gas with the molten metal will have led to extensive oxidation, an exothermic process and the additional heat introduced has led to large amounts of metal being melted.

Samples appear to be bent or warped by the laser cutting process. This is most likely due to residual stresses introduced when material at the cutting-edge melts due to the laser and solidifies again, undergoing shrinkage as it does. The edge suffers an overall contraction by this process, and residual stresses are introduced, causing the centre of the part to deflect, creating a bend in the sample. The cut-off strips (the material removed during the process) demonstrate equal and opposite bending action, which supports this hypothesis, as the effect would occur in reverse when the cut edge is in the centre of the strip.

### 4.3 Electro-magnetic performance

One of the major factors dictating the quality of a stator is the ability of the material to be magnetised, and thus create torque in an electric machine. An experiment was conducted to evaluate the effect of stack cutting on the performance of laminate materials used in stator stacks. Samples were created by laser cutting 7-laminate thick stacks. An Epstein frame was used to evaluate the performance of the samples. The samples were separated into stack position, where position 1 is the top layer (the first layer penetrated by the laser) and 7 is the bottom layer. The layers from position 7 (at the bottom of the stack during cutting) are not used in this test as the samples contain burrs (from the dripping effect) and are therefore unsuitable for use in an Epstein frame.

**Fig 7:**
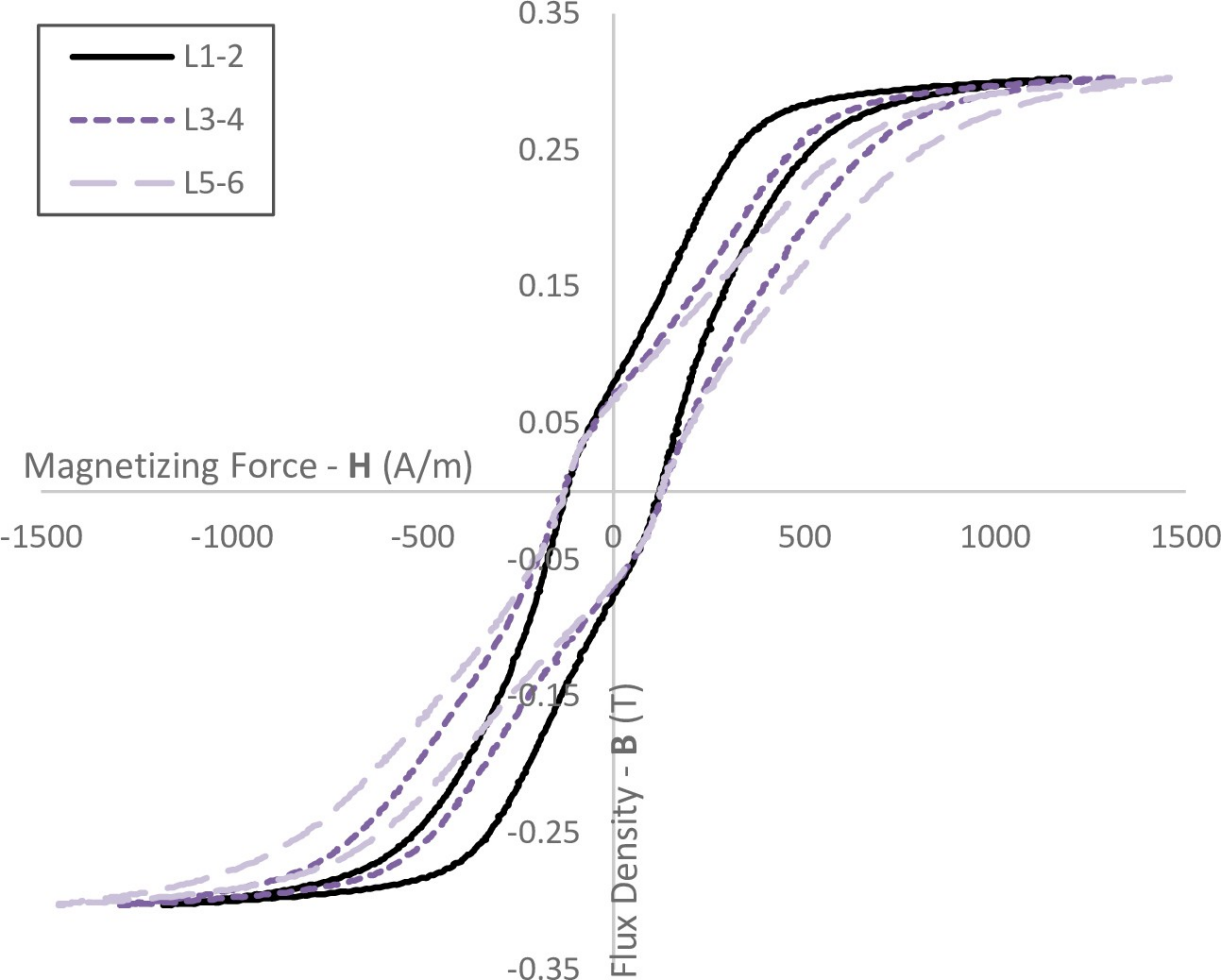
B-H (Flux Density & Magnetizing Force) curve for samples produced by laser cutting stacks.

The results show a reduction in performance for all laser cut samples. There is a clear trend, where samples in lower layers have worse performance than samples in higher layers. The magnetic flux, H, is a desirable feature which directly relates to the torque performance of an electric machine. Fig 7 clearly shows that more magnetising force, B, is required to create the same levels of magnetic flux for samples in layers 3–4, and more so again for layers 5–6.

**Fig 8:**
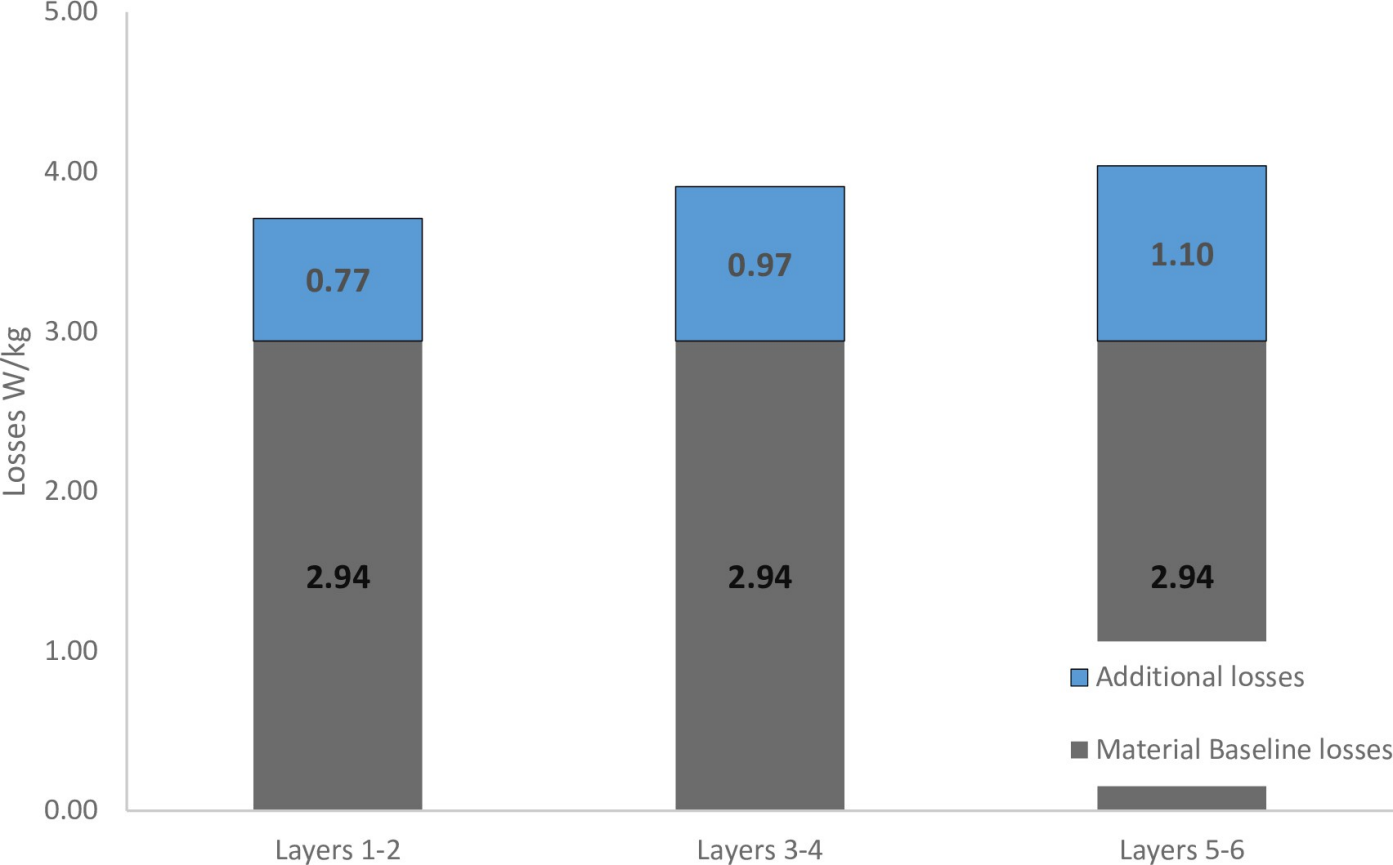
Absolute losses of laminations.

The core losses in the samples are summarised in Fig 8, which shows the total losses as a combination of baseline material losses and additional losses. Additional losses occur in all layers. The additional losses are greater in layers 3–4 and then again in layers 5–6. As the data are limited to this one specific configuration for polystromata cutting, it is difficult to know whether losses would continue to increase for a thicker stack, or if there is a tailing-off of losses through further layers. While the apparent effect on the material microstructure as a result of laser cutting (through the change in grain structure investigated earlier) appears relatively small, it is likely that there is a modification of the magnetic domain structure or quality (features not visible in the SEM) due to the heat introduced by the laser, or by the presence of residual stresses after cutting, which all act to reduce the magnetic performance of the strips cut. In order to fully validate the use of laser cutting in electric machines, the amelioration of these increased losses by further refinement of the processing would need to be investigated.

**Fig 9:**
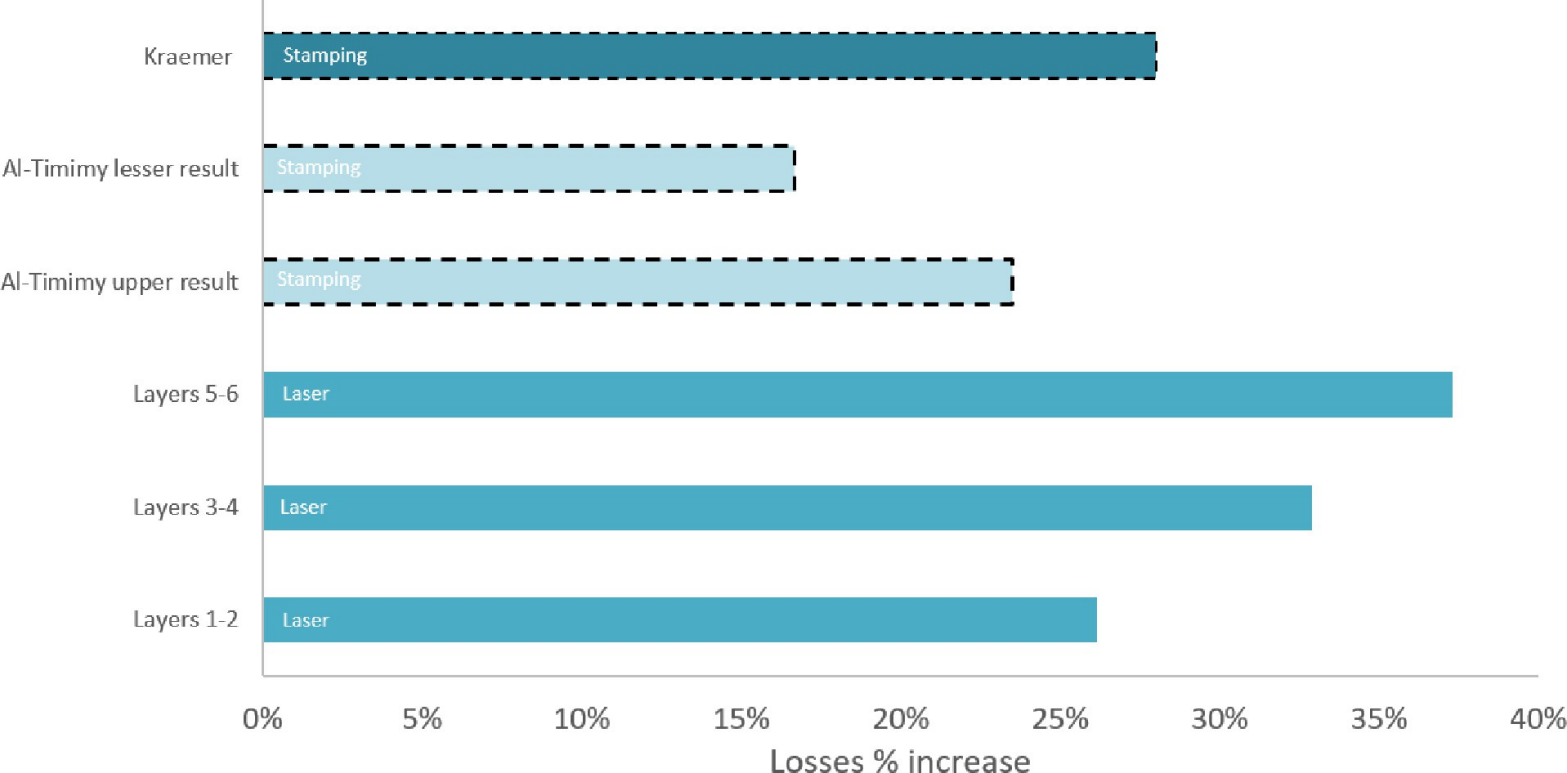
Percentage increase in losses of laminations after different cutting procedures.

It is important to note that other methods of producing laminates also increase the core losses compared to uncut material, though quantitative investigations of these effects are not widespread. Fig 9 shows additional losses as a percentage increase from the baseline material losses. The data previously identified from Al-Timimy [18] shows that the increase in losses is comparable to (but slightly greater than) stamping. Kraemer’s [19] research indicated the relative effect of stamping as an increase in losses compared to losses which would naturally be incurred through the material is 28%.

The current results would suggest that some optimisation of the laser cutting parameters is required from a quality and performance perspective. Given that these results for laser cut samples are based on a new, innovative approach, the comparability of performance in this instance (noting that the studies available to compare the data too are not numerous) may indicate that with further work, greater levels of quality can be produced which could potentially exceed the current standards of stamping.

## 5 Conclusions

Laser cutting can be performed to cut multiple layers simultaneously, as in the polystromata cutting method, and has the potential to improve the productivity of thin sheet metal cutting operations. However, the effectiveness of the process for use in the manufacture of electrical machine components is potentially limited by a reduction in the electro-magnetic performance of the materials.

It has been shown that it is possible to produce a cut for a stack of material in a single cutting operation. The results of the polystromata cuts produced in this study demonstrate that it is possible to achieve a good edge quality and performance relative to stamped materials. Some stacks, particularly larger ones, were found to have a production defect where layers became joined. In most cases the layers could be manually separated post cutting operation, and an additional process would be required as a result. However, in the case of the 23-layer stack, the joining between layers was of much greater strength, and as such it was much more difficult to separate layers post cut. Another issue occurred in which the parts produced were slightly bent or warped. This appears to be due to residual stresses introduced when material at the cutting-edge melts due to the laser and solidifies again, undergoing shrinkage during the process.

As with other cutting methods, laser cutting laminates causes a reduction in laminate magnetic performance. The core losses from laser cut laminates are comparable to those found in stamped samples. Where tested, stamped stators generally have increases in losses as a result of manufacturing in the range 17% - 28% [8, 20, 21] whereas the results of this study indicate additional losses as a result of laser cutting are in the region of 26%-37%. The comparability of performance in this instance may indicate that with further work (investigating process optimisation with different laser cutting equipment), greater levels of quality can be produced with laser cutting, potentially exceeding the current standards of stamping.

To undertake this optimisation, as well a broader investigation of laser cutting conditions and parameters, further development of the methodology could be explored. For example, it might be that multi-pass cutting (where the laser is deflected multiple times around the cut to cause more gradual material removal may be more effective, and reduce some of the material change due to reduced process intensity. This would impact on the efficiency of the process, but the effect would be small as the movements of the laser are rapid, with material movement being a large part of the cycle time.

Laser cutting remains an interesting alternative to stamping and the polystromata method has the potential to be a more effective production method in terms of cost, time, and quality. However, the process requires further work and optimisation to overcome the limitations which were identified in this study; the physical joining of layers, the bending of material, the dripping effect on the bottom layer of a cutting stack. If the process is then optimised further to produce parts with a higher magnetic performance, which is currently comparable to stamping, then polystromata laser cutting will be not only a viable alternative, but a preferred alternative to stamping.
